# Primary signet ring cell carcinoma of the male urethra

**DOI:** 10.3332/ecancer.2022.1426

**Published:** 2022-07-07

**Authors:** Danny Darlington Carbin Joseph, Jagatheswaran Chinnathambi, Arunkumar Jamburaj

**Affiliations:** Department of Urology, Pondicherry Institute of Medical Sciences, Pondicherry, India

**Keywords:** adenocarcinoma, signet ring cell neoplasm, urethral cancer

## Abstract

Malignant tumours of the male urethra are rare and commonly present at an advanced stage. The most common type of urethral cancer is urothelial carcinoma, followed by squamous cell carcinoma. Less than 5% of urethral cancers are adenocarcinoma. We report a young male with signet ring cell carcinoma of the membranous urethra who presented with recurrent lower urinary tract symptoms. Although he was initially treated for a urinary tract infection, the correct diagnosis was made with an appropriate imaging workup. He underwent radical urethrectomy with total pelvic exenteration with bilateral extended pelvic lymph node dissection. Ileal conduit and colostomy were performed as urinary and bowel diversions, respectively. The patient received adjuvant chemotherapy, however, succumbed to COVID-19 8 months after the procedure. This case has been presented to highlight the high index of suspicion required to diagnose this rare malignancy.

## Introduction

Urethral cancer (UC) is an exceedingly rare malignancy occurring more commonly in males. The incidence is 4.3 per million men versus 1.5 per million in females, with a predilection for older men [1]. UC causes symptoms only in the advanced stage. Therefore, delayed presentation is not uncommon. Urothelial carcinoma is the most common subtype of UC, followed by squamous cell carcinoma and adenocarcinoma. Adenocarcinoma contributed less than 5% of all UC reported so far [2]. The male urethra is an unusual location for adenocarcinoma and all cases are reported among females. To the authors’ knowledge, this is the first case of signet ring cell adenocarcinoma of the male urethra reported in the English literature. Although the symptoms were not sinister in the first instance, mimicking urinary tract infection, the tumour was subsequently diagnosed and treated successfully.

## Case report

A 45-year-old man was referred to the outpatient department with lower urinary tract symptoms (LUTS) persisting for over 3 months. He had frequency and difficulty in voiding which was insidious in onset. He denied having hematuria or prior urolithiasis. He did not have any comorbid illnesses and denied any previous urological surgery. He was evaluated in another hospital for LUTS with an ultrasonogram of the kidney, ureters and bladder region, which was normal. His post-void residue was only 20 mL. Therefore, he was treated with empirical antibiotics for suspected urinary tract infection. However, he underwent suprapubic cystostomy elsewhere when he presented with retention and failed urethral catheterisation. He was referred to our tertiary care hospital for further management. Clinical examination revealed a fit and healthy man with good performance status (Karnofsky Performance Scale Index 100). The abdominal examination was also normal. However, a 2 × 3 cm induration was palpable in the bulbo-membranous urethra, free from the overlying perineal skin. There was no inguinal or generalised lymphadenopathy. A digital rectal examination was carried out, which did not reveal any rectal or prostatic lesion.

**Figure 1.**
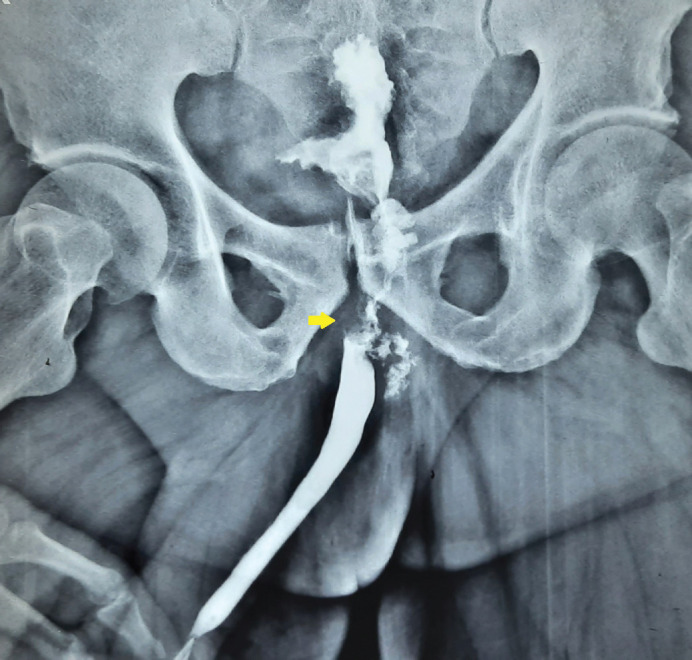
Ascending urethrogram showing the irregular filling defect at the level of bulbo-membranous junction.

**Figure 2.**
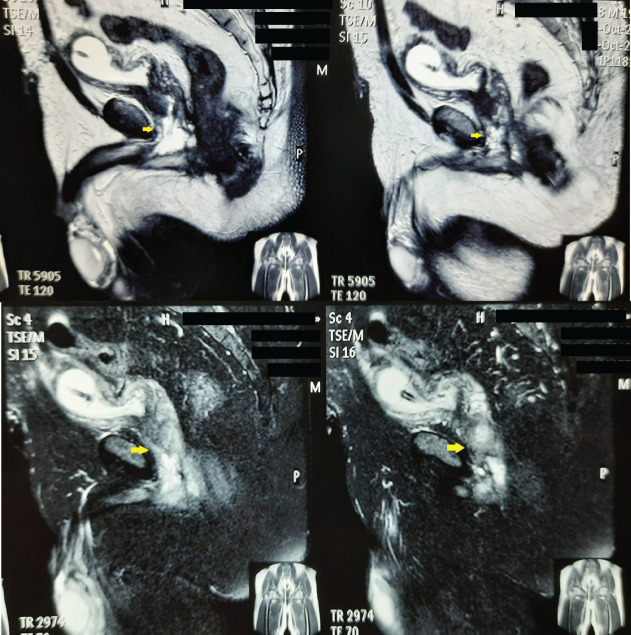
MRI images depicting a T2 hyperintense infiltrative mass involving the membranous urethra.

Laboratory reports including hemogram and renal profile were normal. Serum carcinoembryonic antigen was within the normal limit. The culture of the urine did not reveal any organism. He was subjected to a uroflowmetry which revealed a maximum flow rate of only 8 mL/second. This prompted us to perform a retrograde urethrogram where there was an irregular filling defect at the bulbo-membranous junction with the entry of contrast into the urinary bladder (Figure 1). Given this suspicious finding, a magnetic resonance imaging (MRI) of the abdomen and pelvis was performed with gadolinium-based contrast. The MRI T2 sequence revealed a high signal infiltrative mass of size 2 × 4 cm involving the membranous urethra, extending into the proximal bulbar urethra distally, prostatic urethra proximally and abutting the rectum posteriorly (Figure 2). There was no pelvic lymphadenopathy or ascites, and other abdominal organs were normal. Radiographic examination of the chest was normal. A cystoscopic biopsy was performed under spinal anaesthesia. There was an intraluminal growth with necrotic material at the level of the proximal bulbar urethra beyond which the scope could not be negotiated. Histopathological examination was suggestive of signet ring cell adenocarcinoma of the urethra. Upper gastrointestinal (GI) endoscopy and colonoscopy ruled out any upper GI or colonic primary or infiltration by any rectal tumour.

**Figure 3.**
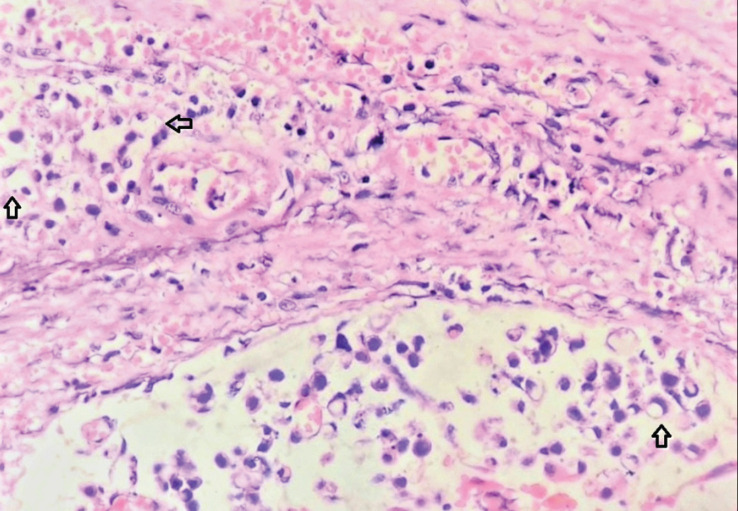
Total pelvic exenteration specimen.

**Figure 4.**
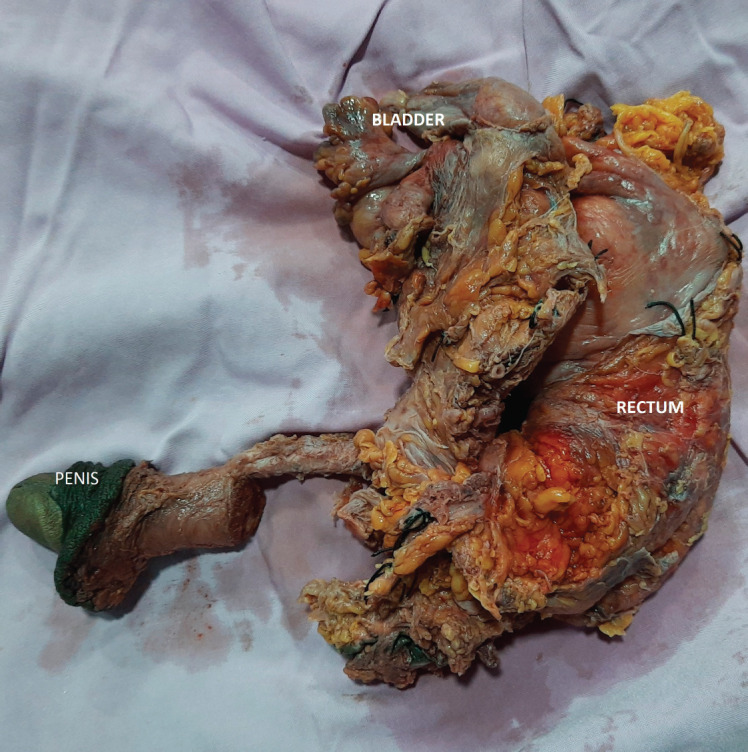
High-power view showing the typical signet ring cells (arrows) with nucleus pushed to the periphery by abundant intracellular mucin (Eosin and Hematoxylin stain, magnification 100×).

After discussing the various options of upfront surgery versus radiotherapy/chemotherapy, the patient opted for surgical treatment and a multi-modality approach. He was planned for open radical cystoprostatectomy and urethrectomy with bilateral extended pelvic lymph node dissection with Ileal conduit diversion. The urethra was approached through the perineum and prepubic and retropubic dissection was performed. The tumour involved the proximal bulbar and membranous urethra with gross extension into the rectum. Therefore, total pelvic exenteration with bilateral extended pelvic lymphadenectomy was performed with ileal conduit and descending colostomy (Figure 3). The postoperative period was uneventful. The suprapubic tract was also excised. Histopathological examination revealed a tumour in membranous urethra composed of lakes of mucin in which clusters of signet ring type carcinomatous cells were visible (Figure 4). Immunohistochemistry showed positivity to β-catenin, carcinoembryonic antigen (CEA) and negative staining to guanine-adenine-thymine-adenine (GATA) 3 and prostate specific antigen (PSA). There was microscopic tumour involvement of the rectal serosa with no mucosal infiltration. In total, 26 pelvic lymph nodes were removed of which none showed evidence of metastases. Given the high-risk primary tumour (pT4N0M0), he was offered Cisplatin-Gemcitabine-based adjuvant chemotherapy after a discussion in the multi-disciplinary treatment. He was doing well on regular follow-ups with no evidence of recurrence after chemotherapy. However, he died of COVID-19-related complications after eight months of surgery.

## Discussion

Primary UC is a rare malignancy occurring more frequently in males than females. The risk factors for UC include long-term catheterisation, chronic infection and inflammation of the urinary tract, stricture urethra, sexually transmitted diseases and prior radiotherapy. Transitional cell carcinoma is the most familiar type of UC followed by squamous cell carcinoma and adenocarcinoma. UC of the prostatic urethra is of transitional type in 90% and squamous in 10% of patients. On the other hand, squamous cell carcinoma contributes to 80%, transitional cell carcinoma to 10% and adenocarcinoma to 10% of UC in the bulbo-membranous urethra [2]. Adenocarcinoma of the urethra arises from the periurethral glands and can be of the following subtypes- enteric, not otherwise specified, mucinous, clear cell and signet ring cell [3].

Primary signet ring cell adenocarcinoma of the urinary tract is extremely rare, with only a handful of cases involving the bladder and upper tracts and female urethra [4–7]. Our case is the first report of signet ring cell adenocarcinoma of the male urethra in the English literature. It was surprising that it occurred in a previously healthy male with no urological risk factor, as discussed above.

UC is notorious for its asymptomatic nature and symptomatic presentation generally implies advanced disease. In previous reports of adenocarcinoma of the male urethra, most presented with symptoms suggestive of bladder outlet obstruction [2, 8]. Our patient presented with uncommon storage symptoms. Therefore, a high index of suspicion was required to arrive at the correct diagnosis. The symptoms and signs of locally advanced disease include palpable mass in the urethra, bladder outlet obstruction, urethra-cutaneous fistulae, periurethral abscess and palpable inguinal nodes. The differential diagnoses such as ductal prostatic adenocarcinoma, transitional cell carcinoma with glandular differentiation, direct invasion or extension from an adjacent organ (rectum or bladder) and metastatic carcinoma should be ruled out before labelling a tumour as primary urethral adenocarcinoma [3]. Signet ring cell adenocarcinoma can arise from the gastrointestinal tract, breast, prostate and lung. Therefore, upper and lower GI endoscopies should be performed to rule out primary tumours in these organs.

The diagnosis of signet ring cell carcinoma of the urethra requires a high degree of suspicion. The importance of a thorough clinical examination cannot be underemphasised. Urine cytology is not reliable in the diagnosis of primary UC. A retrospective study of UC by Touijer *et al* [9] concluded that the urine cytology is not a sensitive test to detect this rare malignancy, in both males and females. Therefore, cystoscopy and biopsy are the best way to diagnose primary UC. MRI is the imaging modality of choice for UC. UC is hypointense in T1 sequence and hyperintense in T2 sequence images [8].MRI also delineates the anatomic extent of the UC, thereby staging the tumour. A chest CT completes the metastatic workup. Under the light microscope, the diagnosis can be confirmed by signet ring cells with cytoplasmic fat vacuoles pushing the nucleus into a peripheral crescent. Immunohistochemistry helps differentiate the rectal versus genitourinary origin of the tumour. Positive staining for β-catenin remains the most useful marker to distinguish these two malignancies [10].Negative staining for GATA3 and absence of urothelial carcinoma in situ ruled out glandular differentiation of urothelial carcinoma [3].While signet ring cell carcinoma of the female urethra stains positively for CEA and negatively for PSA, our case is the first reported male with signet ring cell carcinoma and he had the same pattern of CEA and PSA staining.

Primary signet ring cell carcinoma of the urethra is an extremely rare disease, and therefore, treatment protocols for this malignancy are unclear. The treatment is usually extrapolated from signet ring cell adenocarcinoma occurring in other organs of the genitourinary tract [4, 6]. Distal UC can be treated by penile preserving procedures with or without pelvic lymph node dissection [11].Positive surgical margins, lymphovascular invasion and perineural invasion are risk factors for recurrence after penile preserving surgeries [12].Radical cystoprostatectomy with urethrectomy/penectomy and bilateral pelvic lymph node dissection is the surgical treatment of choice in proximal tumours [12]. However, not uncommonly, patients have a positive surgical margin that necessitates adjuvant chemotherapy. The adjuvant chemotherapy regimens are unclear for this rare malignancy. However, in line with signet ring cell carcinoma of bladder and stomach, both 5-Fluorouracil and Cisplatin-based chemotherapy regimens have been reported. Our patient had good response with Cisplatin-based chemotherapy [4, 13].

Our case report is a rare instance of adenocarcinoma of the male urethra managed with multi-modality treatment. It offers a glimpse into some of the principal treatment options available for this exceedingly rare malignancy. However, being a case report, the management may not be directly extrapolated into other patients with the same disease. This lack of management protocols calls for further studies to understand the disease better and formulate a standardised treatment plan.

## Conclusion

This case has been presented for the rarity of this subtype of UC and the subtle signs and symptoms with which it can present in the clinic. Therefore, a high index of suspicion is required to diagnose it at an early stage of the disease. Persistent LUTS in males cannot always be a recurrent urinary tract infection and, therefore, calls for further evaluation. As UC incidence is extremely low, multi-institutional studies with pooled data will churn out more standardised management protocols.

## Take away message

Signet ring cell adenocarcinoma is a rare malignancy of the male urethra. Persistent LUTS should raise the suspicion of sinister pathology of the urinary tract necessitating further evaluation.

## Conflicts of interest

None.

## Funding

None.

## Author contributions

DD-manuscript writing, data collection, literature search, primary surgeon; JC-manuscript writing, data collection, literature search, images, primary surgical team, AJ-manuscript writing, literature search, primary surgical team, follow up, images.
